# Supplementary material for: Secondary structure prediction for RNA sequences including N6-methyladenosine
Source: Nat Commun. 2022 Mar 11;13:1271. doi: 10.1038/s41467-022-28817-4 (PMC8917230; doi:10.1038/s41467-022-28817-4)
Supplement: Supplementary file 1 — Supplementary Information [file 41467_2022_28817_MOESM1_ESM.pdf]

Supplementary Material for:

Secondary Structure Prediction for RNA Sequences Including N^6-methyladenosine

Elzbieta Kierzek,[1,†] Xiaoju Zhang,[2] Richard M. Watson,[2] Scott D. Kennedy,[2] Marta Szabat,[1] Ryszard Kierzek,[1] and David H. Mathews[2,†]

[1]Institute of Bioorganic Chemistry Polish Academy of Sciences, Noskowskiego 12/14, 61-704 Poznan, Poland

[2]Department of Biochemistry & Biophysics and Center for RNA Biology, 601 Elmwood Avenue, Box 712, School of Medicine and Dentistry, University of Rochester, Rochester, NY 14642

† To whom correspondence should be addressed: David_Mathews@urmc.rochester.edu and Elzbieta.Kierzek@ibch.poznan.pl .

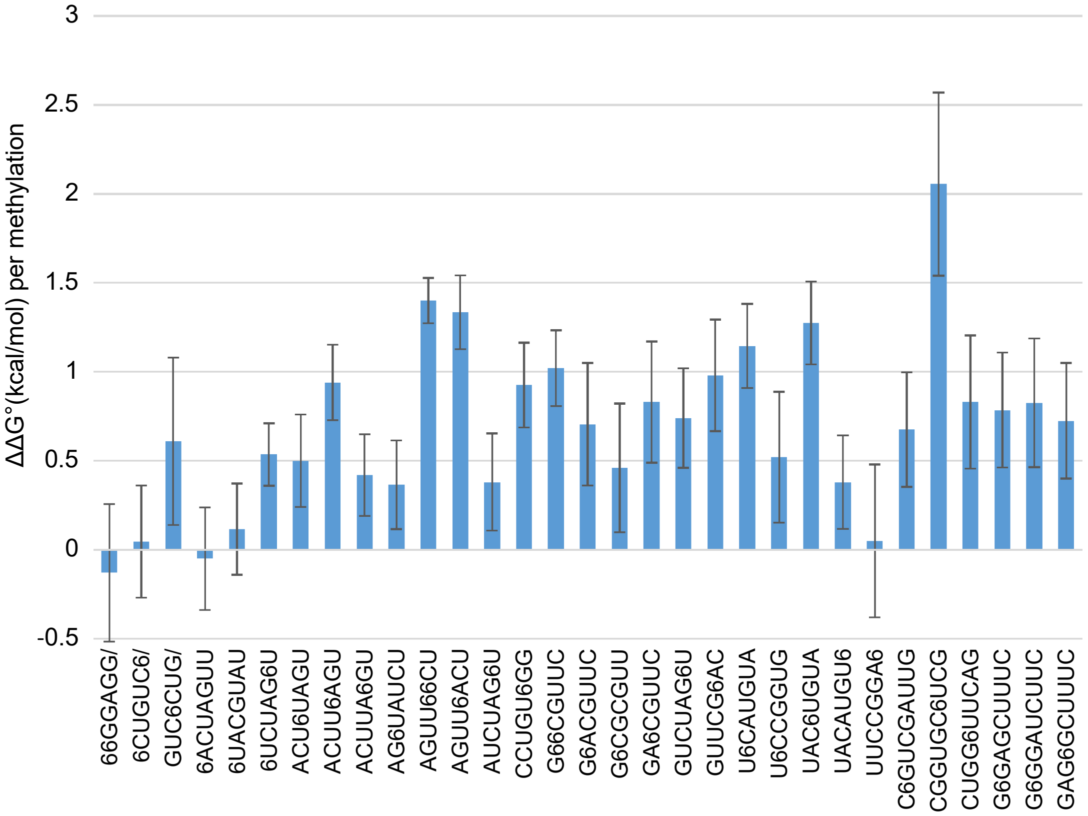

Figure S1. The $\Delta\Delta G^\circ_{37}$ for methylation is sequence dependent. This plot shows the difference in stability per methylation between methylated and unmethylated duplexes, where the positive $\Delta\Delta G^\circ_{37}$ means that the methylated duplex is less stable. 6 indicates the position of m^6A, and sequences ending with / are paired with a complementary strand. The stabilities of the unmethylated duplexes were derived from prior reports[1-6]. Uncertainty is estimated as 4% of the magnitude of free energy from each experiment, as estimated by Xia et al.[3]. The uncertainty in $\Delta\Delta G^\circ_{37}$ derives from error propagation[7].

```
#Description of RNA Folding Alphabet including N6-methyadenosine
#(m6A).

#The Bases field defines the characters allowed in the alphabet.
Bases
X = N = x = n
A = a
C = c
G = g
U = u = T = t
M = m = 6
I

#The Pairing field defines bases that can form canonical pairs.
Pairing
A U
G C
G U
M U

#The Single field defines bases that are not allowed to pair.
Single
a
c
g
u
t
m

#The non-interacting field indicates nucleotides that neither pair nor
#stack.
Non-interacting
X

#The linker field indicates a special "nucleotide" that indicates a
#break in the backbone.
Linker
I
```

Figure S2. The RNAstructure configuration file for the m^6A alphabet. The configuration indicates the nucleotides that are allowed and is read during the initialization of each program. Comments start with "#". The "Bases" field lists the available nucleotides. N or X can be used for bases that are not allowed to pair and provide no stacking stability. U or T can be used for uracil. M or 6 are used to indicate m^6A. I is an intermolecular linker that is used between two stands in bimolecular folding[8]. The "Pairing" field lists the canonical base pairs. "Single" lists nucleotides that cannot form pairs. In RNAstructure, nucleotides in lowercase are not allowed to base pair. "Non-interacting" specifies that N and X provide no stacking stability. Because they are equivalent according to "Bases", only X needs to be specified. "Linker" specifies that "I" is the intermolecular linker, and not an actual part of the sequence.

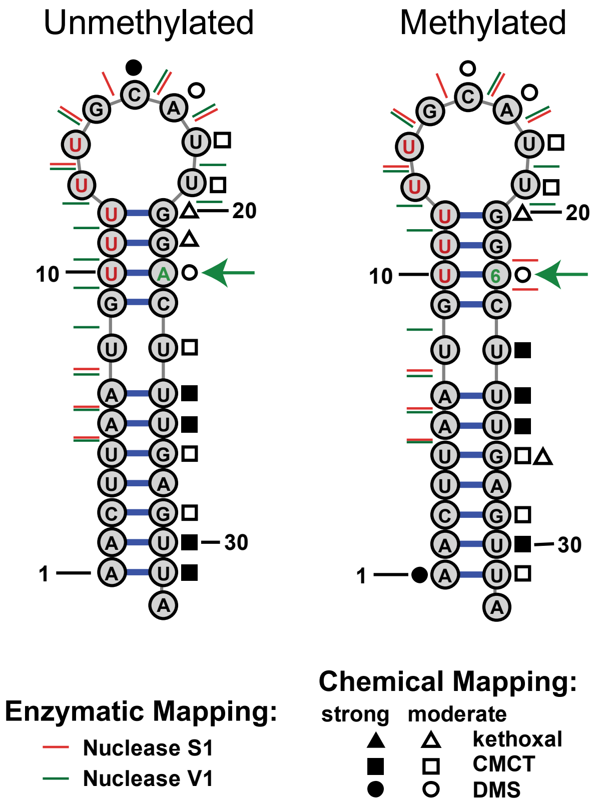

Figure S3. Chemical mapping of MALAT1 hairpin stem-loop. Mapping data for the unmethylated sequence (left) and mapping data for the methylated sequence (right). The chemical agents act on Watson-Crick faces and prefer loop nucleotides, although they also act on helix ends and G-U pairs[9]. Nuclease S1 prefers loop regions and Nuclease V1 prefers helical regions[9]. The nuclease S1 cleavages flanking m^6A for the methylated sequence suggest that the m^6A is more accessible to proteins, although NMR data show that it is paired in methylated and unmethylated sequences (Figures S4 and S8). The reason for the strong CMCT reactivity at U30 is unclear, given the strong imino resonances for U30 that indicate base pairing at 10 °C (Figure S4). It is possible that some end fraying is occurring; the wide imino resonance for U30 at 25 °C (Figure S6) indicates dynamics in the A2-U30 pair.

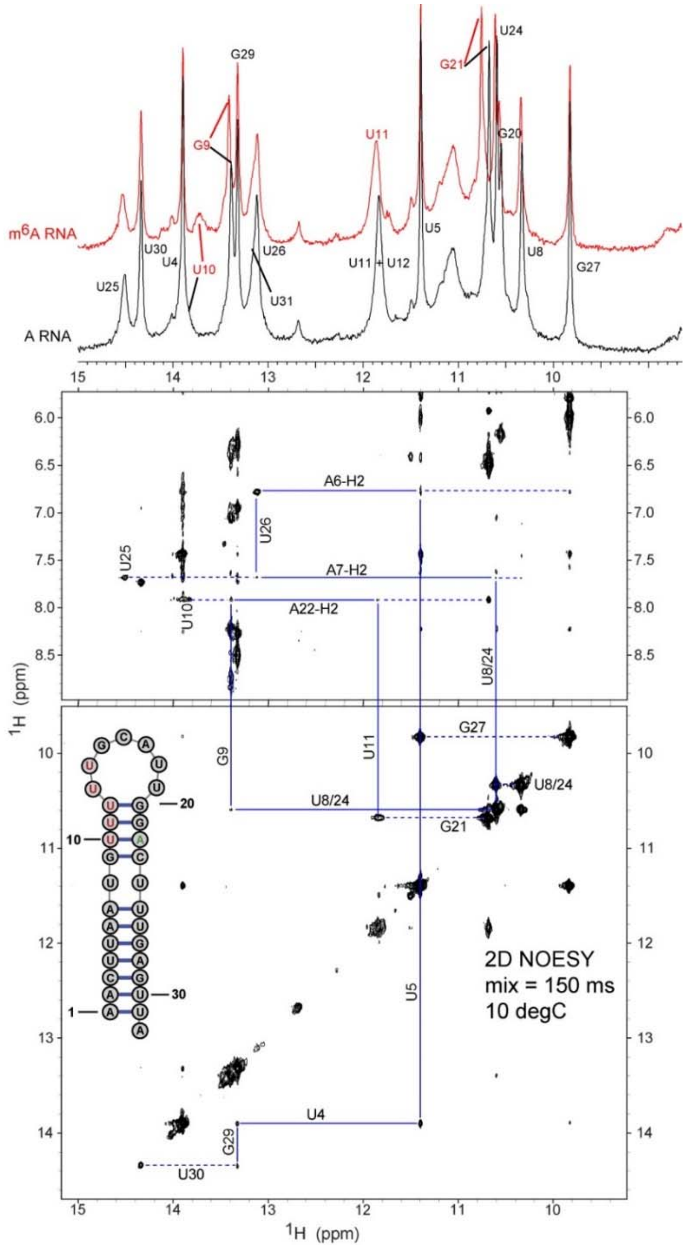

Figure S4. Imino proton region of NMR spectra of MALAT1 hairpin at 10 °C. One dimensional spectra of the unmodified and m^{6}A22-modified sequences are shown above a 2D NOESY spectrum of the unmodified RNA. The 1D spectra show resonance assignments including four shown in red (G9, U10, U11, and G21) that are closest to the methylation site (A22). The sequence of NOESY contacts connected by blue lines show the secondary structure of the unmodified RNA is consistent with the single hairpin (closed conformation) and accounts for all the major peaks in the 1D spectrum. The "walk" shown here makes use of contacts with A6H2, A7H2, and A22H2 instead of directly to U26H3, U25H3, and U10, respectively, because these imino resonances do not exhibit cross-peaks to adjacent base pairs due to solvent exposure. Minor resonances (e.g. 12.7 ppm) are due to a cleavage product. Buffer is 7.5 mM sodium phosphate buffer, pH 6.3, 0.05 mM EDTA. RNA concentrations are 0.75 mM for m^{6}A22-RNA and 0.61 mM A22-RNA. Low RNA and sodium phosphate buffer concentrations were used to limit the extent of duplex formation (Figure S5).

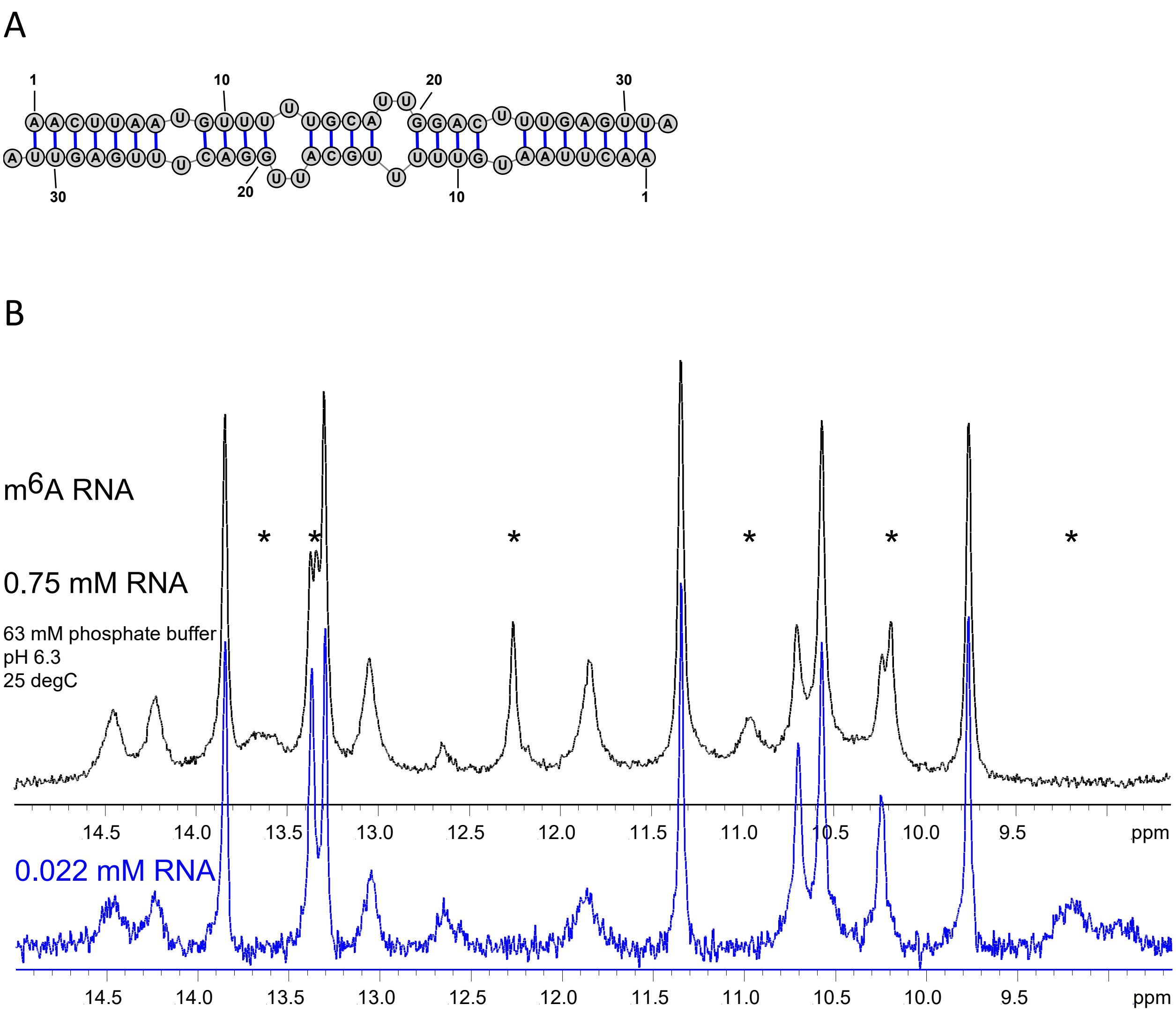

Figure S5. MALAT1 RNA stem-loop sequences formed a duplex at high concentration. At near physiologic counter ion concentrations, the 32 nucleotide MALAT1 RNA used for NMR studies exhibited duplex formation near RNA concentrations required for 2D NMR experiments. Panel A: The presumed duplex structure, where the hairpin loop region in the stem-loop forms canonical base pairs. Panel B: Some peaks (marked with asterisks) at 0.75 mM m^{6}A-RNA in a buffer containing 63 mM sodium phosphate (top) are not observed after 34-fold dilution with the same buffer (bottom). For instance, the peak at 12.25 ppm is due to a G-C pair (G15-C16*) in the duplex. Other (hairpin) peaks are not affected. Consequently, 2D NMR experiments for identifying the hairpin secondary structure were carried out in low sodium phosphate (7.5 mM) to disfavor duplex formation near millimolar RNA concentration (note the lack of signal at 12.25 ppm in 1D spectra in Figure S4).

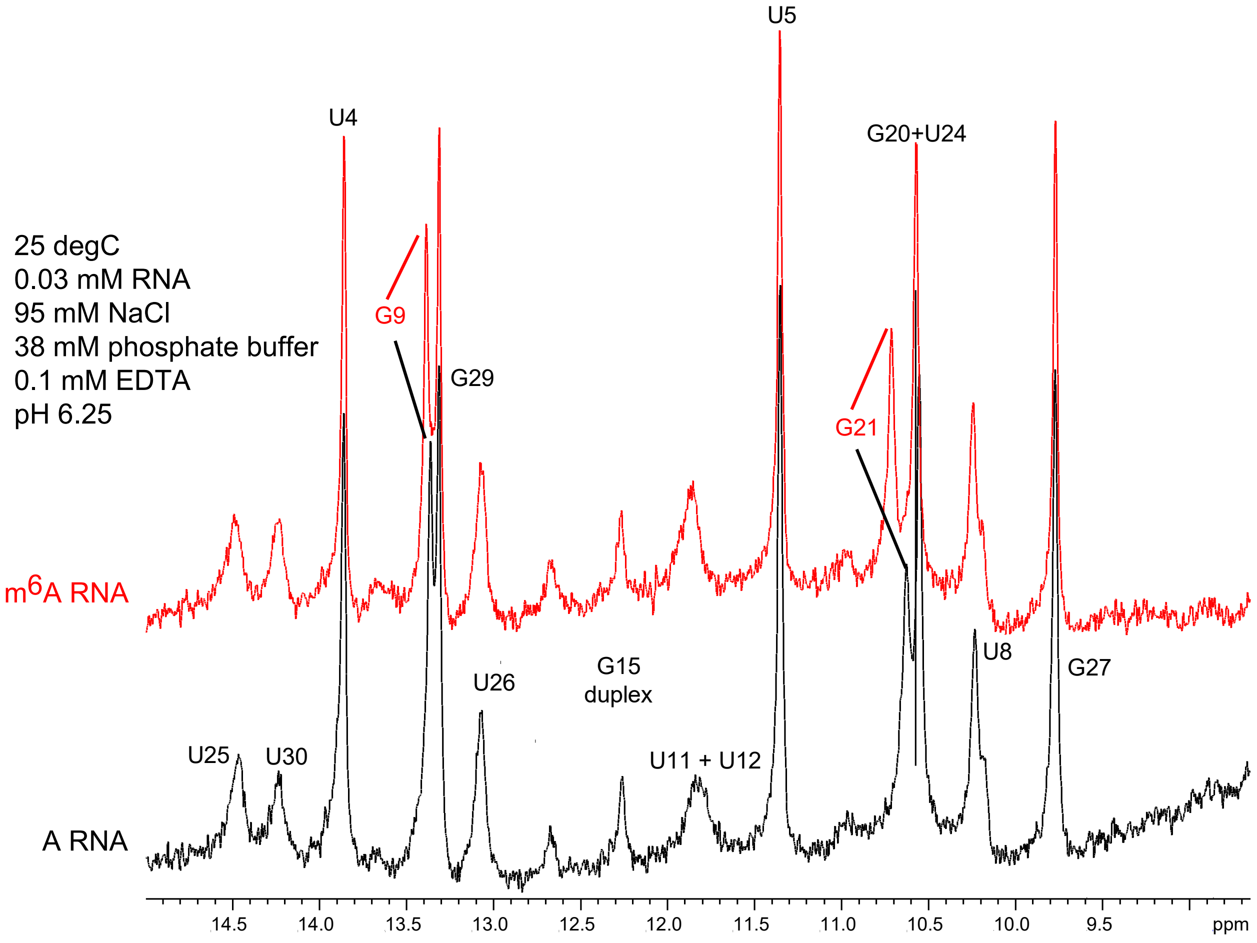

Figure S6. MALAT1 stem-loop NMR spectra are not affected by N^6-methylation in 95 mM NaCl and 38 mM sodium phosphate buffer at 25 °C. A low RNA concentration is used to minimize formation of duplex. As at 10 °C in 7.5 mM sodium phosphate buffer (Figure S4), the only peaks that show significant chemical shift changes are near the methylation site (A22).

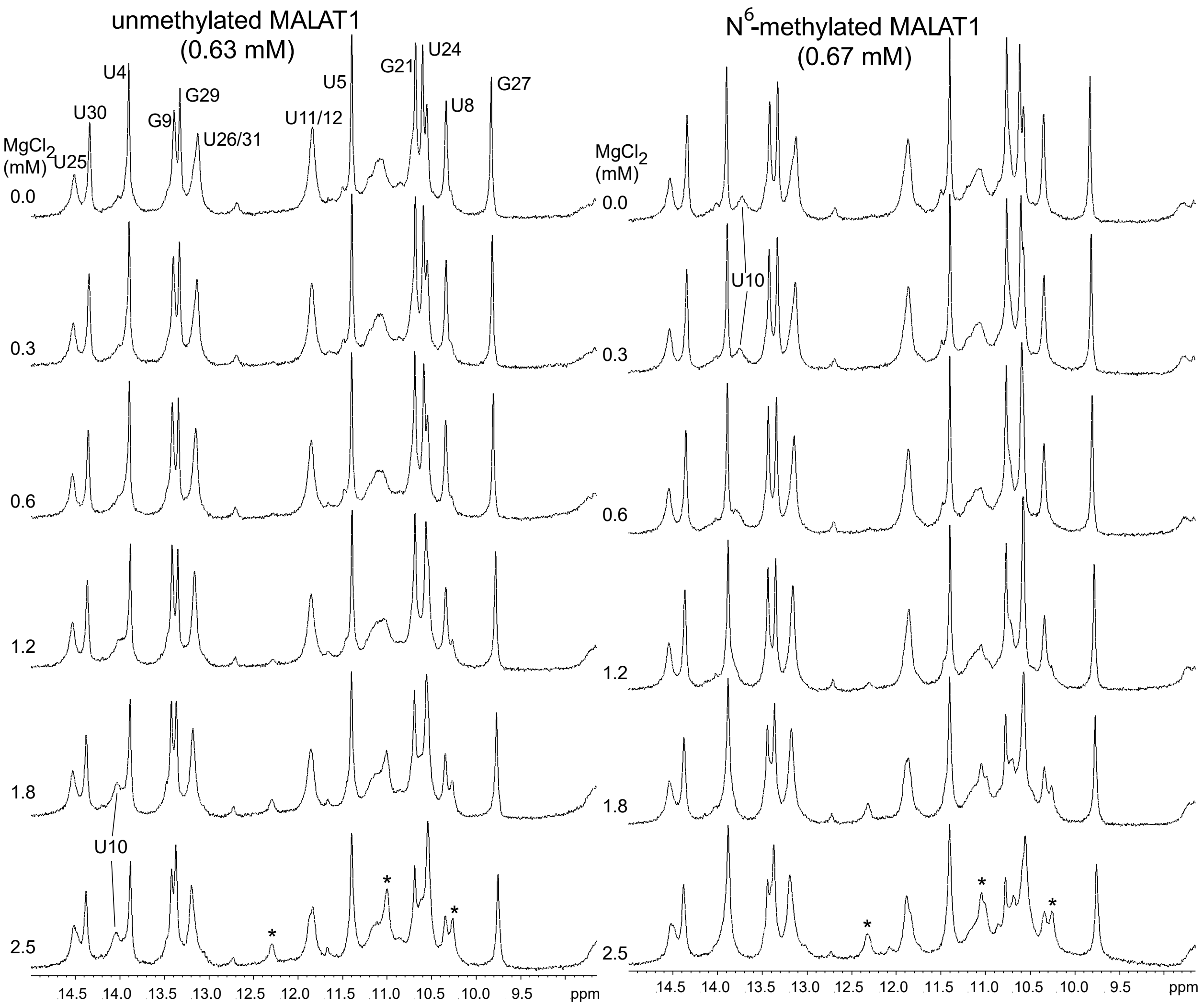

Figure S7. Effect of Mg^{2+} on 1D imino proton NMR spectra of unmethylated (left) and N^6-methylated (right) MALAT1 stem-loop at 10 °C. Starting buffer is 7.5 mM sodium phosphate buffer, pH 6.3, 0.05 mM EDTA. Resolved signals from RNA duplex formed in the presence of Mg^{2+} are indicated with asterisks at the highest Mg^{2+} concentration. The imino signal from residue U10, indicated where it is not overlapped with U4, changes by ~0.2 ppm in both unmodified and methylated RNA due to 2.5 mM added $MgCl_2$. See Figure S8 for NOESY assignment of U10.

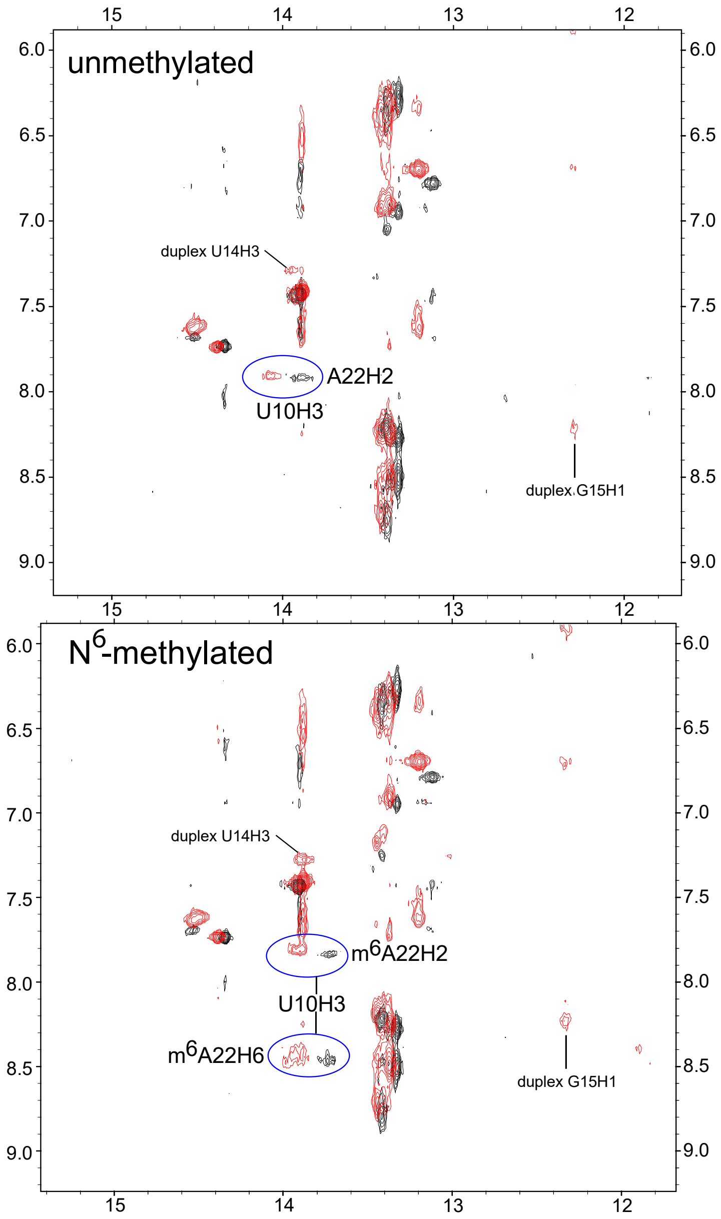

Figure S8. Two-dimensional NOESY spectra in the absence (black; 50 ms mix) and presence (red; 100 ms mix) of 2.5 mM $MgCl_2$ for unmethylated (top) and N^6-methylated (bottom) MALAT1 at 10 °C. Assignment of U10 and A22 signals are highlighted in blue. Cross-peaks from the duplex population that appears with addition of $MgCl_2$ indicate UA and GC pairs assigned to U14 and G15, respectively, in the duplex internal loop. Starting buffer is 7.5 mM sodium phosphate buffer, pH 6.3, 0.05 mM EDTA.

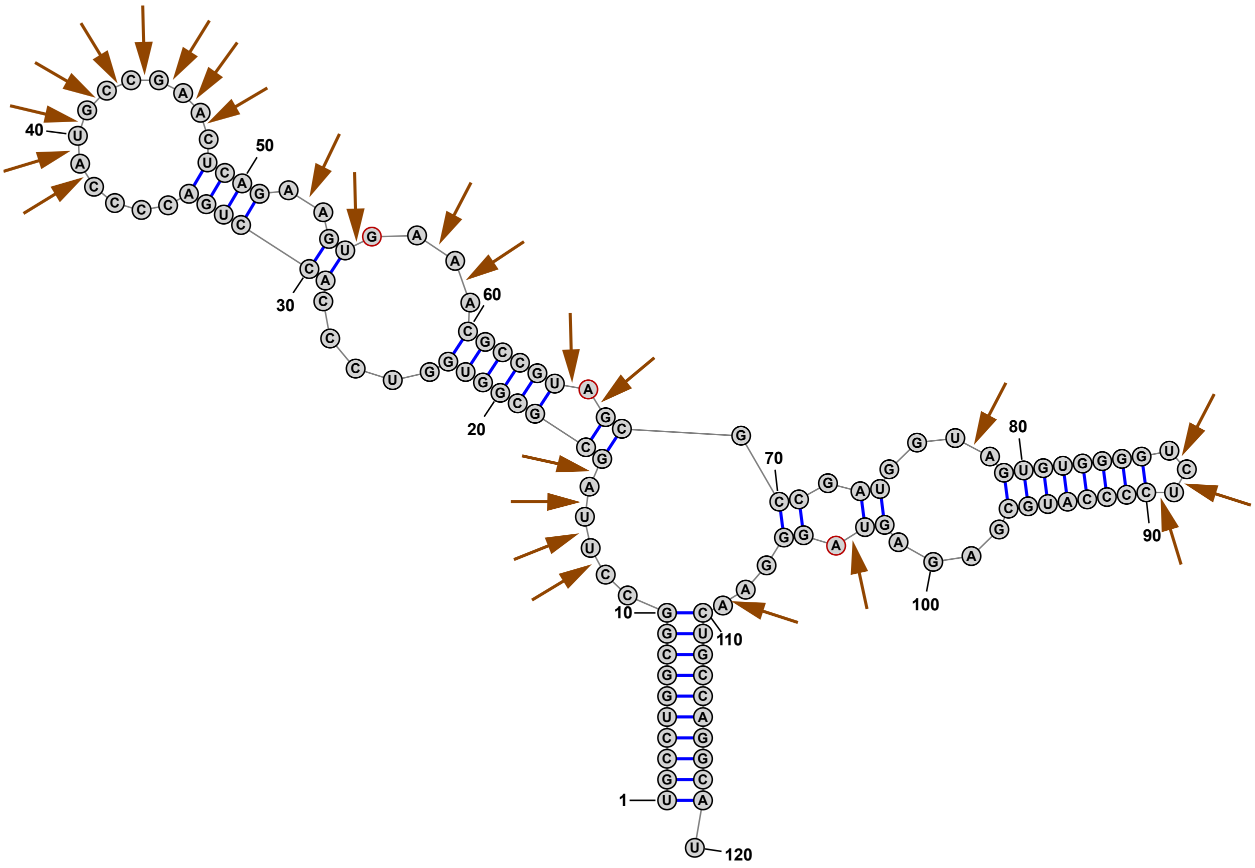

Figure S9. S1 nuclease mapping of 5S rRNA. This shows the data of Speek and Lind[10] superimposed upon the accepted structure of the *E. coli* 5S rRNA[11]. Arrows indicate backbone cleavages by S1 nuclease. The mapping pattern is consistent with cleavages both 5' and 3' to unpaired nucleotides. Nucleotides G56, A66, and A104 are unpaired bases with 5' cleavages where the 5' base is paired. This demonstrates that a subset of PARS S1 cleavages attributed to the 5' base being unpaired should be attributed to the 3' base being unpaired.

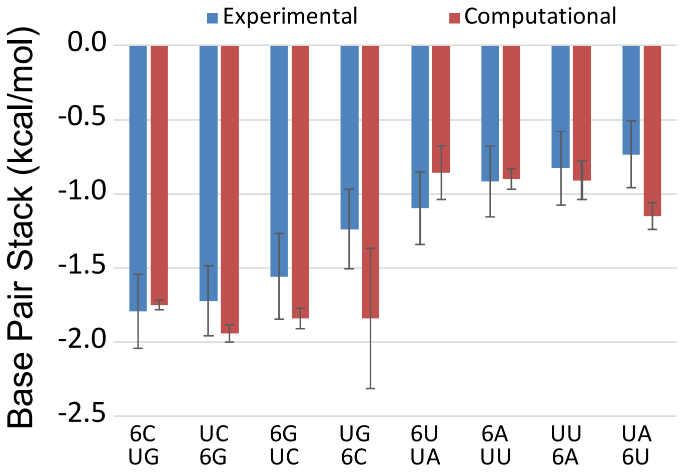

Figure S10. Comparison of computationally-estimated stacking nearest neighbor parameters (red)[12] to our experimentally-determined nearest neighbors (Blue). Overall the agreement of the estimates is excellent. The U-m^{6}A pair followed by a G-C pair has the largest deviation of 0.60 kcal/mol. The uncertainty in the experimentally-determined parameters in the standard error of the regression. The uncertainty in the computationally-estimated parameters is the standard error of the mean from multiple simulations[12].

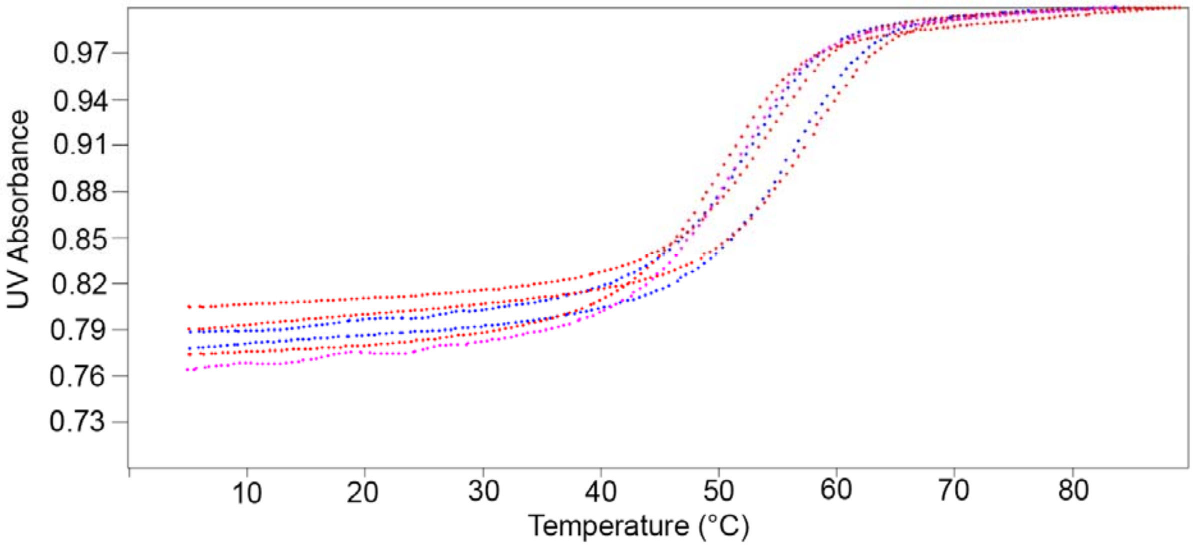

Figure S11A. The optical melting curves for 5’CGGUGCm^{6}AUCG$_{2}$ in 1 M NaCl. From top to the bottom, the total strand concentrations (C_T) are 1.385×10^{-4}, 7.936×10^{-5}, 3.748×10^{-5}, 2.156×10^{-5}, 1.201×10^{-5}, and 7.279×10^{-6} M.

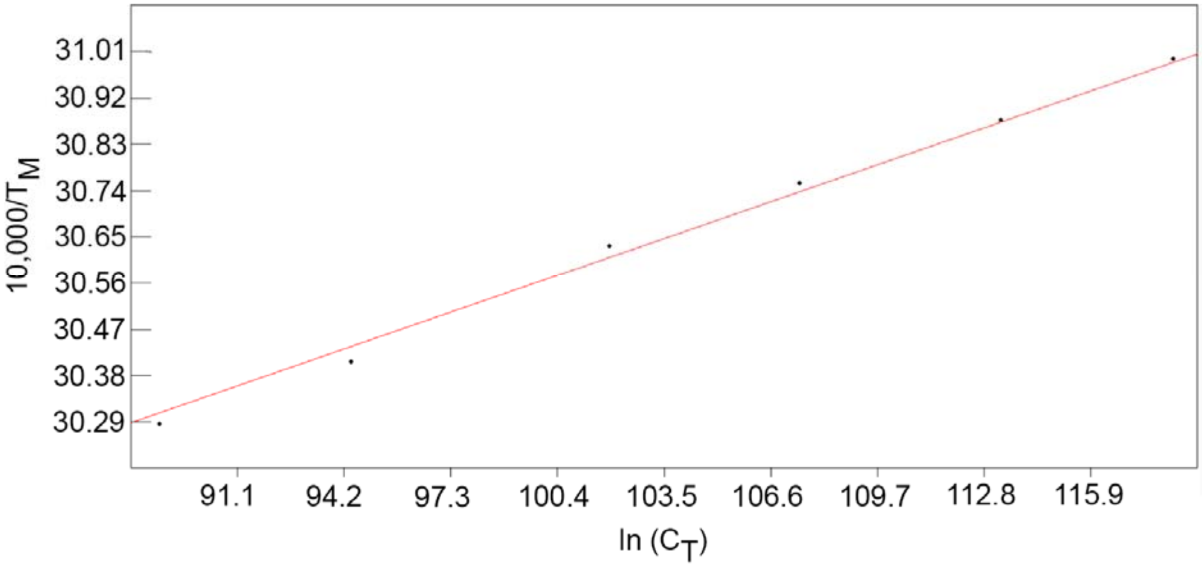

Figure S11B. T_M^{-1} vs. ln(C_T) plot for 5’CGGUGCm^{6}AUCG in 1 M NaCl.

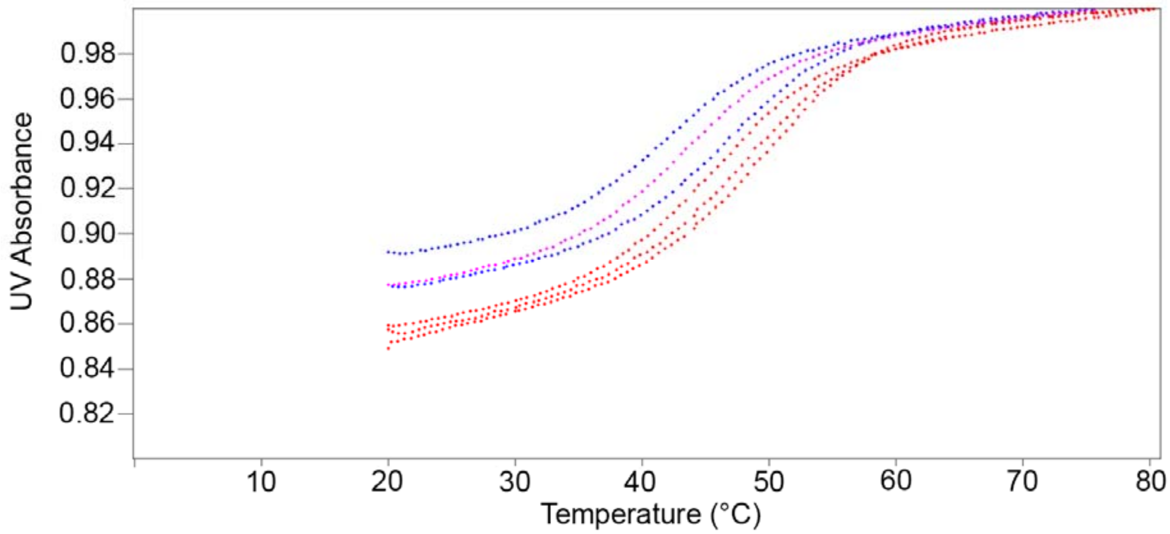

Figure S11C. The optical melting curves for 5'GGCAGm^{6}ACUC/3'CCGCUGAG in 1 M NaCl. From top to the bottom, the total strand concentrations (C_T) are 9.816×10^{-5}, 4.517×10^{-5}, 2.974×10^{-5}, 2.064×10^{-5}, 1.305×10^{-5}, and 7.769×10^{-6} M.

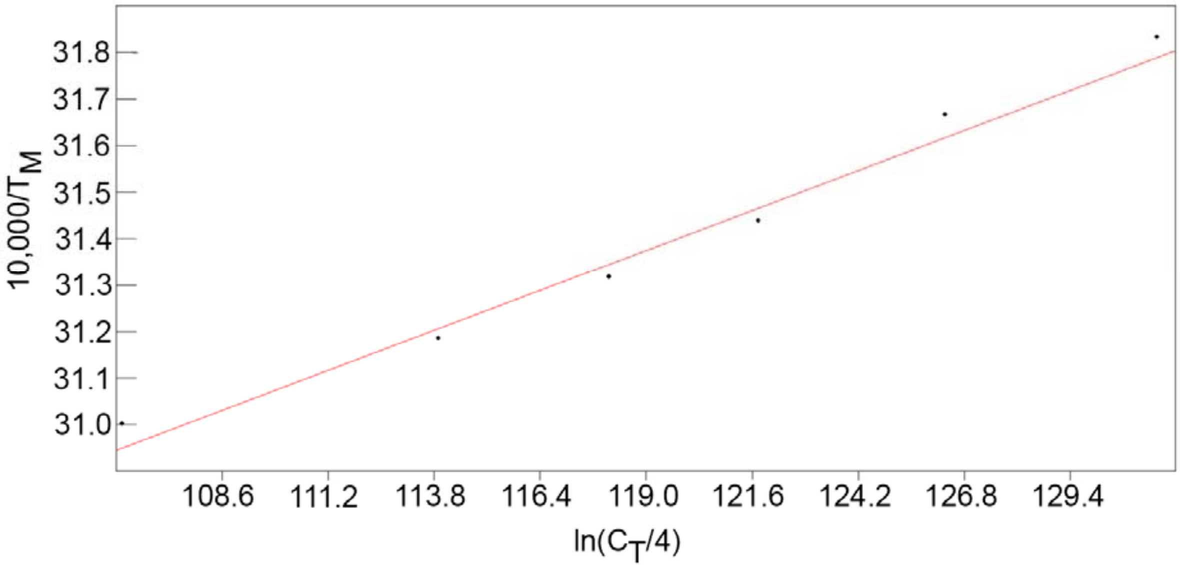

Figure S11D. T_M^{-1} vs. ln(C_T/4) plot for 5'GGCAGm^{6}ACUC/3'CCGCUGAG in 1 M NaCl.

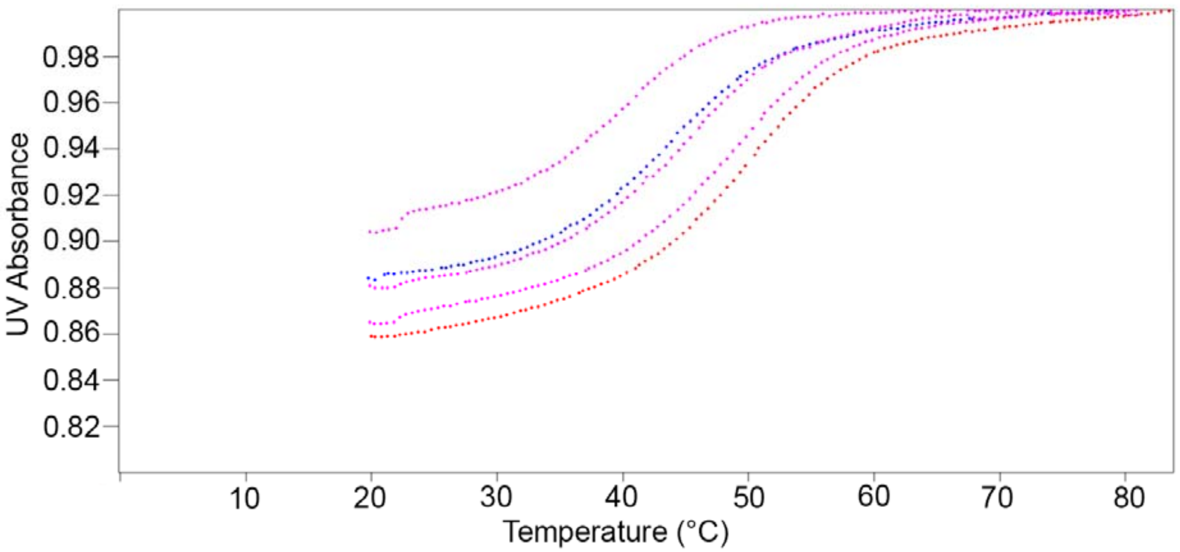

Figure S11E. The optical melting curves for 5'GGCAGm⁶ACUC/3'CCGCUGAG in 150 mM KCl and 5 mM $MgCl_2$. From top to the bottom, the total strand concentrations (C_T) are 1.791×10^{-4}, 1.190×10^{-4}, 2.580×10^{-5}, 1.708×10^{-5}, and 5.324×10^{-6} M.

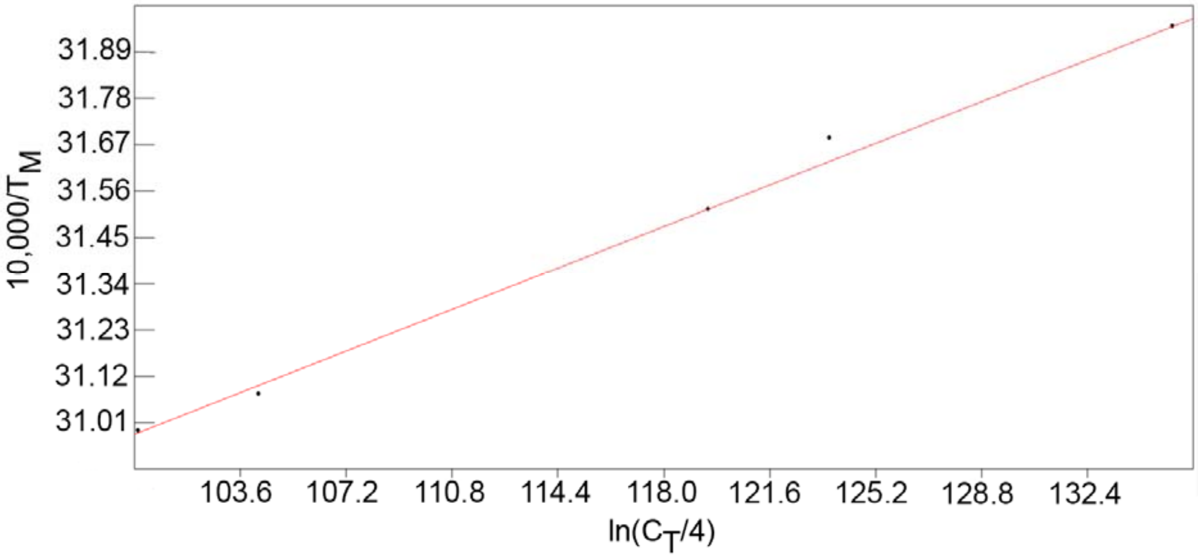

Figure S11F. T_M^{-1} vs. $\ln(C_T/4)$ plot for 5'GGCAGm⁶ACUC/3'CCGCUGAG in 150 mM KCl and 5 mM $MgCl_2$.

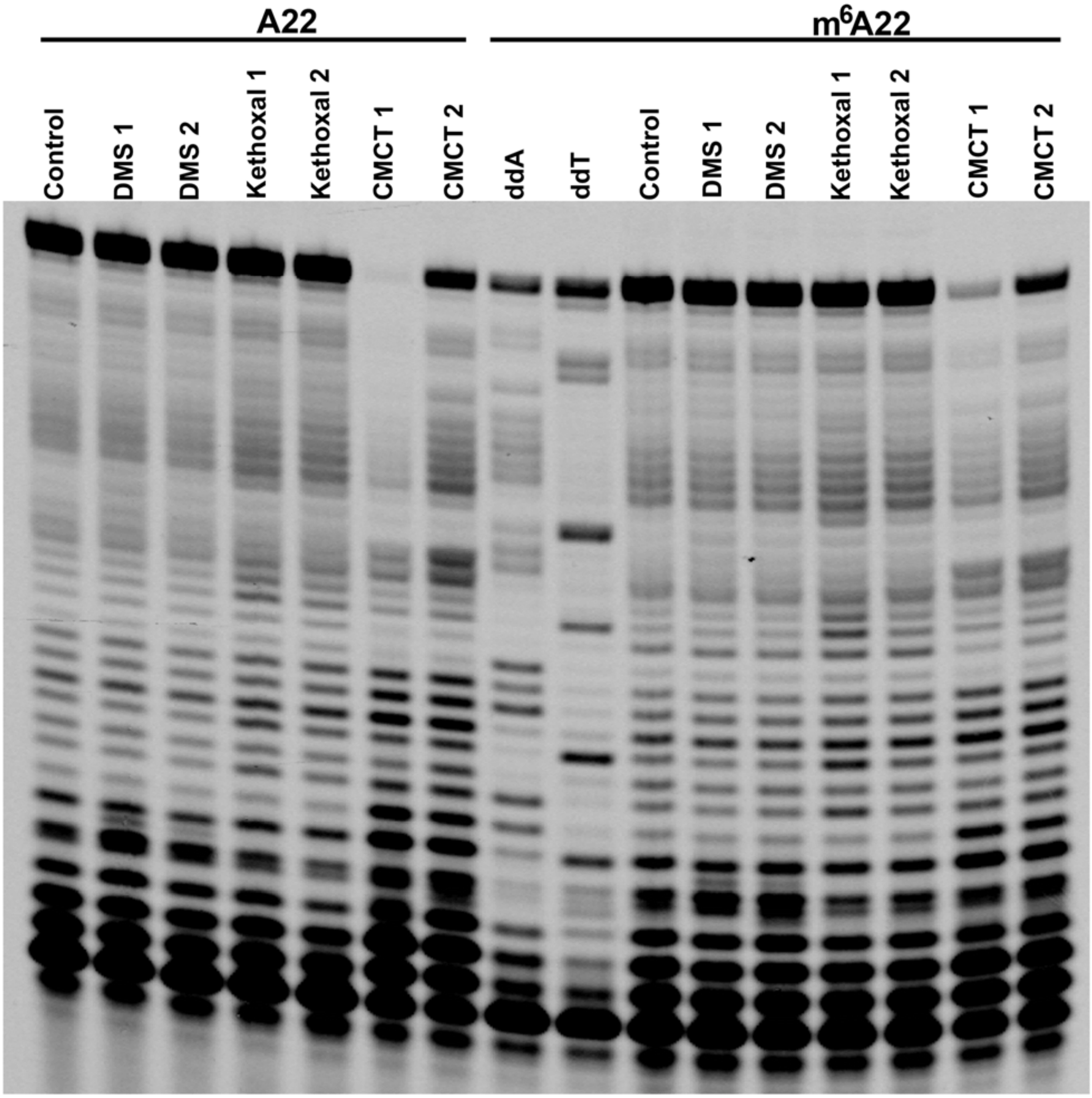

Figure S12. Chemical mapping gel image. The mapping experiments were performed twice with each reagent for the methylated and unmethylated hairpins. The ddA and ddT lanes are dideoxy-sequencing lanes to indicate the position in the hairpin stem-loop. The control experiment, in the absence of mapping reagent, is on the left.

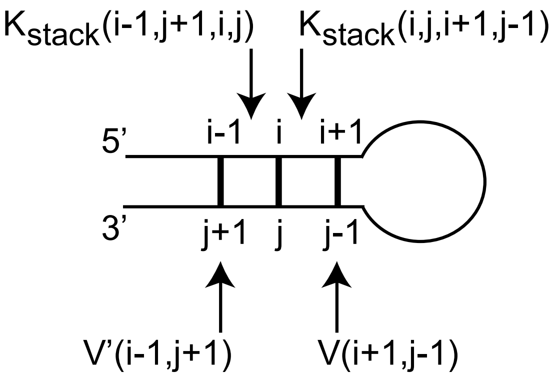

Figure S13. The calculation that the i-j base pair is buried in a helix. In the illustration, the base pair i-j is stacking between adjacent pairs, (i+1)-(j-1) and (i-1)-(j+1). The contribution to the partition function for this configuration is the product of four terms: K_{stack}(i-1,j+1,i,j), the equilibrium constant for the stack of two base pairs; K_{stack}(i,j,i+1,j-1), the equilibrium constant for the other base pair stack; V(i+1,j-1), the partition function from nucleotides i+1 to j-1, inclusive, given that i+1 and j-1 base pair; and V'(i-1,j+1), the partition function from nucleotides 1 to i and also j to N (the length of the sequence), given that i-1 and j+1 base pair. The calculations for V and V' are provided by the recursions in Mathews as implemented in RNAstructure[13], and follow the algorithm of McCaskill[14]. The total probability that nucleotide i is in a base pair that is stacked on adjacent base pairs in determined by summing these probabilities for all j to which nucleotide i can base pair.

Table S1. Helical duplexes used to fit stacking nearest neighbor parameters. This table provides the results of the two-state fits to optical melting data: the folding enthalpy change (ΔH°), the folding entropy change (ΔS°), and the folding free energy change at 37 °C ($\Delta G°_{37}$) determined from the enthalpy and entropy changes. If the enthalpy changes of the average of the curve fits and the analysis of T_M dependence agree to within 15%, the melting is consistent with two-state behavior.

	Analysis of T_M Dependence			Average of the Curve Fits		
Sequence†:	$\Delta G°_{37}$ (kcal/mol)	ΔH° (kcal/mol)	ΔS° (cal mol^{-1} K^{-1})	$\Delta G°_{37}$ (kcal/mol)	ΔH° (kcal/mol)	ΔS° (cal mol^{-1} K^{-1})
66GGAGG/ UUCCUCC	-9.80±0.28	-61.3±7.0	-166.3±22.1	-9.75±0.42	-55.2±8.5	-146.5±26.5
6CUGUC6/ UGACAGU	-7.83±0.01	-46.6±1.4	-125.0±4.5	-7.87±0.08	-51.1±2.1	-139.5±6.6
GUC6CUG/ CAGUGAC	-8.01±0.04	-61.0±2.4	-170.8±7.8	8.06±0.12	-61.8±4.5	173.3±14.4
6ACUAGUU$_2$	-7.26±0.10	-54.7±4.5	-153.0±14.3	-7.23±0.16	-51.1±2.2	-141.7±7.0
6UACGUAU$_2$	-6.30±0.04	-40.9±1.6	-111.8±5.5	-6.31±0.20	-45.3±4.9	-125.7±16.1
6UCUAG6U$_2$	-5.06±7.7	-52.3±2.3	-152.3±7.7	-5.08±0.05	-52.5±3.0	-153.0±10.0
ACU6UAGU$_2$	-5.98±0.20	-38.0±4.5	-103.2±14.9	-5.96±0.20	-43.6±5.4	-121.5±16.9
ACUU6AGU$_2$	-4.28±0.21	-45.1±4.5	-131.7±15.2	-4.32±3.7	-45.3±3.7	-132.1±12.3
ACUUA6GU$_2$	-5.32±0.08	-49.1±2.7	-141.3±8.9	-5.36±0.24	-50.8±4.6	-146.6±15.6
AG6UAUCU$_2$	-5.85±0.08	-47.4±2.2	-134.1±7.4	-5.71±0.10	-52.5±1.8	-151.1±5.6
AGUU66CU$_2$	-0.76±0.41	-54.8±4.0	174.5±14.2	-1.96±0.67	-43.5±5.6	-134.0±20.2
AGUU6ACU$_2$	-3.69±0.25	-45.3±3.7	-134.1±12.7	-3.95±0.22	-42.0±4.0	-122.9±13.4
AUCUAG6U$_2$	-6.44±0.04	-56.9±3.4	-162.7±11.0	-6.42±0.16	-50.5±3.0	-142.2±9.6
CCUGU6GG$_2$	-4.96±0.17	-49.8±4.1	-144.6±13.7	-4.93±0.12	-53.7±7.6	-157.5±24.7
G66CGUUC$_2$	-5.22±0.15	-54.9±4.8	-160.3±15.8	-5.28±0.10	-51.8±6.1	-150.1±19.8
G6ACGUUC$_2$	-7.89±0.08	-75.9±3.9	-219.4±12.3	-7.68±0.16	-63.8±5.1	-181.2±16.7
G6CGCGUU$_2$	-8.58±0.04	-61.1±1.1	-169.5±3.6	-8.40±0.16	-56.8±3.6	-156.1±11.3
GA6CGUUC$_2$	-7.64±0.06	-57.4±2.9	-160.5±8.1	-7.85±0.14	-66.4±3.8	-188.7±12.1
GGUU6ACC$_2$	-6.64±0.06	-51.7±3.4	-145.3±11.1	-6.63±0.08	-55.6±3.8	-158.1±12.2
GUCUAG6U$_2$	-6.22±0.02	-52.6±1.7	-149.6±5.8	-6.24±0.08	-54.5±2.6	-155.6±8.4
GUUCG6AC$_2$	-6.80±0.01	-61.0±1.8	-174.9±5.7	-6.83±0.09	-62.0±2.5	-178.1±8.1
U6CAUGUA$_2$‡	-4.67±0.04	-55.7±1.3	-164.6±4.2	4.75±0.13	54.8±3.8	161.4±12.5
U6CCGGUG$_2$	-8.66±0.06	-49.9±1.6	-133.0±5.1	-8.68±0.08	-49.5±3.8	-131.5±12.4
UAC6UGUA$_2$‡	-4.41±0.07	-57.2±1.6	-170.2±5.4	4.36±0.24	58.9±5.5	175.8±18.3
UACAUGU6$_2$‡	-6.20±0.01	-57.5±1.4	-165.4±4.6	6.27±0.05	56.8±5.1	163.0±16.5
UUCCGGA6$_2$	-10.69±0.13	-65.6±2.0	-177.1±6.2	-10.58±0.18	-172.5±7.6	-64.1±2.5
C6GUCGAUUG$_2$	-7.35±0.02	-58.9±1.9	-166.3±6.2	-7.37±0.08	-60.9±1.4	-172.7±5.0
CGGUGC6UCG$_2$	-10.65±0.11	-86.0±2.5	-243.2±7.8	-10.60±0.12	-84.5±2.2	-238.5±6.8
CUGG6UUCAG$_2$	-8.49±0.18	-77.4±7.0	-222.2±22.0	-8.38±0.07	-72.3±1.7	-205.0±5.4
G6GAGCUUUC$_2$	-7.25±0.01	-63.7±1.5	-182.0±4.8	-7.23±0.06	-62.8±1.5	-179.2±4.7
G6GGAUCUUC$_2$	-8.18±0.11	-66.8±4.1	-189.1±13.0	-8.14±0.11	-65.7±2.0	-185.6±6.3
GAG6GCUUUC$_2$	-7.37±0.07	-76.8±4.6	-223.7±14.7	-7.39±0.14	-77.9±1.8	-227.2±5.9

† m^6A is indicated as 6.

‡Reported previously[5].

Table S2. Nearest neighbor stacks for m^6A-U pairs. M^6A is indicated with 6

Nearest Neighbor Stack:	$\Delta G°_{37}$ (kcal/mol)
5’6C3’<br>3’UG5’	-1.79±0.25
5’UC3’<br>3’6G5’	-1.72±0.24
5’6G3’<br>3’UC5’	-1.56±0.29
5’UG3’<br>3’6C5’	-1.24±0.27
5’6U3’<br>3’UA5’	-1.10±0.25
5’6A3’<br>3’UU5’	-0.92±0.24
5’UU3’<br>3’6A5’	-0.83±0.25
5’UA3’<br>3’6U5’	-0.73±0.23
5’6U3’<br>3’UG5’	-0.69±0.32
5’6U3’<br>3’U65’	-0.46±0.36
5’UG3’<br>3’6U5’	-0.32±0.33
5’UU3’<br>3’6G5’	-0.32±0.27
5’663’<br>3’UU5’	-0.21±0.34
5’6G3’<br>3’UU5’	-0.03±0.29
5’U63’<br>3’6U5’	+1.45±0.57

.

Table S3. Stabilities of dangling end, terminal mismatch, and loop sequences with m^6A (shown as "6"). Unpaired nucleotides are underlined.

		Analysis of T_M Dependence			Average of Curve Fits		
Sequence:	Motif:	$\Delta G°_{37}$ (kcal/mol)	$\Delta H°$ (kcal/mol)	$\Delta S°$ (cal mol^{-1} K^{-1})	$\Delta G°_{37}$ (kcal/mol)	$\Delta H°$ (kcal/mol)	$\Delta S°$ (cal mol^{-1} K^{-1})
ACAUGU6 †,‡<br>6UGUACA	3' Dangling End	-5.79±0.02	-62.0±1.8	-181.3±5.9			
GCGC6 †<br>6CGCG	3' Dangling End	-8.89±0.04	-52.4±0.9	-140.4±2.8	-8.78±0.04	-50.1±0.09	-133.2±2.9
CCGG6 †<br>6GGCC	3' Dangling End	-7.77±0.07	-51.9±1.8	-142.2±5.7	-7.73±0.22	-49.5±4.4	-134.8±13.6
6AUGAAU †<br>UACGUA6	5' Dangling End	-6.49±0.02	-46.8±1.8	-130.1±6.0	-6.51±0.12	-47.4±3.2	-131.9±10.3
6GCGC6 †<br>6CGCG6	Terminal Mismatch	-8.21±0.21	-42.6±3.8	-111.0±11.7	-8.39±0.26	-45.4±3.4	-119.3±10.3
6UGCGCA6 †<br>6ACGCGU6	Terminal Mismatch	-9.92±0.21	-54.5±3.0	-143.7±8.9	-9.91±0.34	-54.7±3.8	-144.4±11.2
6UGCGC66 †<br>66CGCGU6	Terminal Mismatch	-9.70±0.26	-51.7±3.6	-135.3±10.6	-9.81±0.27	-53.4±3.1	-140.5±9.1
6UGCGCAG †<br>GACGCGU6	Terminal Mismatch	-9.74±0.15	-51.4±2.1	-134.3±6.5	-10.23±0.20	-58.5±2.6	-155.6±7.9
CGGCC6 †<br>6CCGGC	Terminal Mismatch	-9.06±0.19	-50.4±2.9	-133.2±8.7	-9.20±0.22	-52.2±1.8	-138.7±5.1
6GGCGCUG †<br>GUCGCGG6	Terminal Mismatch	-10.86±0.49	-68.6±6.4	-186.2±19.0	-10.24±0.27	-60.4±3.2	-161.8±9.7
GAUGCAU6 †<br>6UACGUAG	Terminal Mismatch	-7.29±0.09	-54.3±3.4	-151.6±10.7	-7.37±0.12	-59.2±3.1	-167.0±9.9
GGCGU6AUAGCC	Hairpin Loop	N.A. [A]	N.A. [A]	N.A. [A]	-3.19±0.11	-33.3±1.1	-97.3±3.5
GCGG6UGC †<br>CGU6GGCG	Internal Loop	-4.30±0.01	-44.6±0.12	-129.9±0.4	-4.25±0.04	-45.4±0.7	-132.6±2.5

†These are self-complementary duplexes. Both strands in duplexes are shown to demonstrate the motifs.

‡ This 3' dangling end was previously measured[5].

A. Hairpin loops are unimolecular and therefore the T_M is not concentration-dependent.

Table S4. Loop motif stabilities where 6 indicates m^{6}A. The $\Delta\Delta G^\circ_{37}$ is the stability of the motif with m^{6}A minus the stability of the analogous motif with A; therefore, $\Delta\Delta G^\circ_{37} < 0$ indicates m^{6}A is more stabilizing than an analogous A. The determination of the motif stability is detailed in the Methods in equation 1.

Motif Sequence:	Motif Type:	Reference ΔG°_{37} (kcal/mol)	Motif m^{6}A ΔG°_{37} (kcal/mol)	Motif A ΔG°_{37} (kcal/mol)	Motif $\Delta\Delta G^\circ_{37}$ (kcal/mol)
U6<br>A	3' Dangling End	-5.28±0.30 [A]	-0.43±0.20	-0.21±0.19 [B]	-0.22±0.27
C6<br>G	3' Dangling End	-4.61±0.18 [C]	-2.14±0.20	-1.66±0.18 [D]	-0.49±0.27
G6<br>C	3' Dangling End	-4.55±-0.18 [E]	-1.61±0.18	-1.15±0.16 [F]	-0.47±0.24
6A<br>U	5' Dangling End	-4.42±0.33 [A]	-1.04±0.21	-0.48±0.20 [G]	-0.56±0.29
C6<br>G6	Terminal Mismatch	-4.61±0.18 [C]	-1.80±0.18	-1.52±0.18 [H]	-0.29±0.26
A6<br>U6	Terminal Mismatch	-8.22±0.33 [I]	-0.85±0.26	-0.73±0.25 [J]	-0.12±0.36
66<br>U6	Terminal Mismatch	-7.16±0.61 [A]	-1.27±0.36	-0.73±0.25 [J]	-0.54±0.51
AG<br>U6	Terminal Mismatch	-8.22±0.33 [I]	-0.76±0.25	-0.78±0.26 [J]	0.02±0.36
C6<br>GC	Terminal Mismatch	-5.37±0.21 [K]	-1.85±0.21	-1.47±0.20 [H]	-0.38±0.29
UG<br>G6	Terminal Mismatch	-8.42±0.34 [L]	-1.22±0.27	-0.48±0.25 [M]	-0.74±0.37
U6<br>AG	Terminal Mismatch	-4.42±0.33 [A]	-1.44±0.22	-1.23±0.21	-0.18±0.31
CGU6AUAG	Hairpin Loop	-6.68±0.11 [A]	3.49±0.17	3.26±0.17 [N]	0.23±0.24
GG6U<br>U6GG	Internal Loop	-4.82±0.33 [O]	0.52±0.37	0.19±0.38 [P]	0.33±0.53

A. Estimated with nearest neighbor parameters[3].
B. Analogous A-containing duplex measured previously[5].
C. Reference was measured previously[15].
D. Analogous A-containing duplex measured previously[16].
E. Reference was measured previously[17].
F. Analogous A-containing duplex measured previously[17].
G. Analogous A-containing duplex measured previously[18].
H. Analogous A-containing duplex measured previously[19].
I. Reference was measured previously[16].
J. Analogous A-containing duplex measured previously[20].
K. Reference was measured previously[21].
L. Reference was measured previously[22].
M. Analogous A-containing duplex measured previously[23].
N. Analogous A-containing hairpin measured previously[24].
O. Reference duplex estimated with nearest neighbor parameters[6].
P. Analogous A-containing duplex measured previously[25].

Table S5A. Additional optical melting experiments to test nearest neighbor model. 6 is used to represent m^6A. - indicates the position of a bulge loop in the strand without the bulged nucleotide. These optical melting experiments were performed with 1 M NaCl.

	Analysis of T_M Dependence			Average of the Curve Fits		
Sequence:	$\Delta G°_{37}$ (kcal/mol)	$\Delta H°$ (kcal/mol)	$\Delta S°$ (cal mol^{-1} K^{-1})	$\Delta G°_{37}$ (kcal/mol)	$\Delta H°$ (kcal/mol)	$\Delta S°$ (cal mol^{-1} K^{-1})
$(GGACUAGUCC)_2$	-16.19±0.56	-95.0±5.7	-254.2±16.8	-16.99±0.12	-103.2±0.9	-278.2±2.8
$(GG6CUAGUCC)_2$	-15.36±0.75	-101.7±8.1	-273.4±23.9	-15.54±0.22	-103.6±1.9	-283.9±5.5
$(CGCGCG)_2$	-9.38±0.14<br>(9.11±0.05)†	-47.8±1.9<br>(54.4±0.5)†	-124.0±5.9<br>(146.2±1.6)†	-10.26±0.36<br>(9.06±0.14)†	-59.8±2.2<br>(53.1±2.0)†	-159.9±6.3<br>(-142.1±6.1)†
$(CGCGCG6)_2$	-12.25±0.22	-68.3±2.4	-180.7±7.0	-12.19±0.12	-67.6±0.9	-178.7±2.6
CAGAGACUCG<br>GUCU-UGAGC	-8.74±0.16	-61.2±5.1	-169.1±16.3	-8.70±0.21	-57.0±4.3	-155.9±13.4
CAGAG6CUCG<br>GUCU-UGAGC	-8.00±0.24	-47.7±7.1	-127.8±22.7	-8.01±0.16	-54.3±1.4	-149.1±4.0
CAGGCACUCG<br>GUCCG-GAGC	-12.27±0.49	-76.1±7.3	-205.7±22.0	-11.23±0.43	-60.3±4.7	-158.3±13.9
CAGGC6CUCG<br>GUCCG-GAGC	-11.90±0.31	-76.6±5.2	-208.3±15.8	-11.60±0.51	-69.6±7.1	-187.2±21.4
GGCGGACUC‡<br>CCGC-UGAG	-10.37±0.16	-65.5±3.4	-177.8±10.3	-10.74±0.32	-73.1±3.8	-200.9±11.2
GGCGG6CUC‡<br>CCGC-UGAG	-9.88±0.15	-67.2±3.6	-184.9±11.2	-10.05±0.23	-71.0±3.4	-196.7±10.2
GGCAGACUC‡<br>CCG-CUGAG	-9.78±0.46	-66.4±8.9	-182.4±27.7	-9.96±0.11	-71.4±0.9	-198.3±2.9
GGCAG6CUC‡<br>CCG-CUGAG	-9.07±0.22	-66.0±6.2	-183.6±19.2	-9.20±0.12	-70.4±1.5	-197.5±4.5
GCAUGA-CUGC‡*<br>UGUACUAGACG	-11.7±0.35	-89.7±6.8	-251.5±20.8	-11.84±0.26	-92.8±2.9	-261.0±8.8
GCAUG6-CUGC‡*<br>UGUACUAGACG	-10.75±0.12	-89.1±3.1	-252.5±9.6	-10.55±0.13	-82.5±5.1	-238.0±16.1
GGCGGACUC‡<br>CCGCC-GAG	-13.05±0.58	-86.5±8.5	-236.8±25.7	-12.63±0.60	-80.5±8.8	-218.7±26.6
GGCGG6CUC‡<br>CCGCC-GAG	-11.76±0.35	-70.0±5.0	-187.9±15.1	-11.73±0.42	-69.7±5.6	-187.0±16.9

†Previous measurement of $CGCGCG_2$ with a 3' terminal phosphate, measured by Freier et al.[15] and reported by SantaLucia et al.[26].

‡ These sequences are duplexes that contain bulge loop motifs studied previously in hairpin stem-loops by Liu et al. (2018).[27] We studied these as duplexes to obtain concentration-dependent optical melting data (Analysis of T_M Dependence) and to reduce the T_M in 1 M NaCl and in 150 mM KCl and 5 mM $MgCl_2$ (Table S5B).

*This loop was designed previously by Liu et al. (2018)[27] to mimic a structure found in hepatitis C virus[28].

Table S5B. Additional optical melting experiments to test nearest neighbor model using $MgCl_2$. 6 is used to represent m^6A. - indicates the position of a bulge loop in the strand without the bulged nucleotide. These optical melting experiments were performed with 150 mM KCl and 5 mM $MgCl_2$.

	Analysis of T_M Dependence			Average of the Curve Fits		
Sequence:	$\Delta G°_{37}$ (kcal/mol)	$\Delta H°$ (kcal/mol)	$\Delta S°$ (cal mol^{-1} K^{-1})	$\Delta G°_{37}$ (kcal/mol)	$\Delta H°$ (kcal/mol)	$\Delta S°$ (cal mol^{-1} K^{-1})
$(GG6CUAGUCC)_2$	-13.57±0.37	-88.2±4.4	-240.5±13.1	-14.66±0.98	-100.9±10.6	-278.2±30.9
GGCGGACUC<br>CCGC-UGAG	-10.73±0.14	-76.1±3.0	-210.6±9.3	-10.97±0.21	-81.7±4.9	-227.9±15.4
GGCGG6CUC<br>CCGC-UGAG	-10.26±0.06	-76.2±1.7	-212.7±5.4	-10.32±0.34	-75.8±7.5	-211.3±9.3
GGCAGACUC<br>CCG-CUGAG	-9.63±0.16	-75.5±5.2	-212.3±16.2	-9.54±0.14	-72.2±3.2	-202.1±9.9
GGCAG6CUC<br>CCG-CUGAG	-8.75±0.18	-68.5±6.0	-192.7±19.2	-8.76±0.15	-68.8±3.0	-194.2±9.4
GCAUGA-CUGC<br>UGUACUAGACG	-11.23±0.28	-97.8±6.9	-279.0±21.5	-11.30±0.27	-99.5±3.5	-284.5±10.7
GCAUG6-CUGC<br>UGUACUAGACG	-9.88±0.10	-91.2±3.5	-262.2±10.9	-9.61±0.12	-81.1±4.1	-230.5±12.9
GGCGGACUC<br>CCGCC-GAG	-12.04±0.67	-71.3±9.1	-191.1±27.3	-12.35±0.61	-75.4±7.8	-203.4±23.2
GGCGG6CUC<br>CCGCC-GAG	-11.42±0.36	-68.7±5.6	-184.7±16.8	-11.60±0.25	-71.6±2.2	-193.4±6.4

Table S6A. Accuracy of nearest neighbor estimates. 6 stands for m^{6}A. - indicates the position of a bulge loop in the strand without the bulged nucleotide.

Sequence:	Experimental ΔG°$_{37}$ (kcal/mol)	Estimated ΔG°$_{37}$ (kcal/mol)	Error in Estimate (ΔΔG°$_{37}$; kcal/mol)
(GGACUAGUCC)$_2$	-16.19±0.56	-16.67±0.35	0.48±0.73
(GG6CUAGUCC)$_2$	-15.36±0.75	-14.51±0.76	-0.85±0.97
(CGCGCG)$_2$	-9.38±0.14	-9.40±0.38	0.02±0.54
(CGCGCG6)$_2$	-12.25±0.22	-12.26±0.68†	0.01±0.84
CAGAGACUCG<br>GUCU-UGAGC	-8.74±0.16	-8.60±0.69‡	-0.14±0.78
CAGAG6CUCG<br>GUCU-UGAGC	-8.00±0.24	-7.05±0.77‡	0.05±0.84
CAGGCACUCG<br>GUCCG-GAGC	-12.27±0.49	-13.02±0.70‡	0.75±0.85
CAGGC6CUCG<br>GUCCG-GAGC	-11.9±0.31	-13.02±0.70‡	1.12±0.85
GGCGGACUC<br>CCGC-UGAG	-10.37±0.41	-10.59±0.69‡	0.22±0.80
GGCGG6CUC<br>CCGC-UGAG	-9.88±0.40	-9.95±0.72‡	0.07±0.82
GGCAGACUC<br>CCG-CUGAG	-9.78±0.39	-10.16±0.69‡	0.38±0.79
GGCAG6CUC<br>CCG-CUGAG	-9.07±0.36	-9.08±0.87‡	0.01±0.94
GCAUGA-CUGC<br>UGUACUAGACG	-11.67±0.47	-11.77±0.71‡	0.10±0.85
GCAUG6-CUGC<br>UGUACUAGACG	-10.75±0.43	-11.14±0.75‡	0.39±0.87
GGCGGACUC<br>CCGCC-GAG	-13.05±0.52	-12.25±0.70‡	-0.80±0.88
GGCGG6CUC<br>CCGCC-GAG	-11.76±0.47	-12.25±0.70‡	0.49±0.85

†The uncertainty estimate for the dangling A increment was taken from Zuber et al.[29].

‡ The uncertainty estimate for the bulge of one nucleotide increment was taken from Zuber et al.[29].

Table S6B. Differences in Folding Stability Between 1 M NaCl and 150 mM KCl with 5 mM $MgCl_2$. 6 stands for m^6A. - indicates the position of a bulge loop in the strand without the bulged nucleotide.

Sequence:	$\Delta G°_{37}$ (kcal/mol) 1 M NaCl	$\Delta G°_{37}$ (kcal/mol) 150 mM KCl 5 mM $MgCl_2$	$\Delta\Delta G°_{37}$ (kcal/mol)	% Difference†
$(GG6CUAGUCC)_2$	-15.36±0.75	-13.57±0.37	-1.79±0.84	-12.3
GGCGGACUC<br>CCGC-UGAG	-10.37±0.16	-10.73±0.14	0.36±0.21	3.4
GGCGG6CUC<br>CCGC-UGAG	-9.88±0.15	-10.26±0.06	0.38±0.16	3.8
GGCAGACUC<br>CCG-CUGAG	-9.78±0.46	-9.63±0.16	-0.15±0.49	-1.5
GGCAG6CUC<br>CCG-CUGAG	-9.07±0.22	-8.75±0.18	-0.32±0.28	-3.6
GCAUGA-CUGC<br>UGUACUAGACG	-11.7±0.35	-11.23±0.28	-0.47±0.45	-4.1
GCAUG6-CUGC<br>UGUACUAGACG	-10.75±0.12	-9.88±0.10	-0.87±0.16	-8.4
GGCGGACUC<br>CCGCC-GAG	-13.05±0.58	-12.04±0.67	-1.01±0.89	-8.1
GGCGG6CUC<br>CCGCC-GAG	-11.76±0.35	-11.42±0.36	-0.34±0.50	-2.9

†% Difference is defined as the $\Delta\Delta G°_{37}$ divided by the mean folding free energy change of the two conditions: $2\times\Delta\Delta G°_{37} / |\ \Delta G°_{37\ Na} + \Delta G°_{37\ K+Mg}|$

Table S7. Chemical shifts of the 32 nucleotide MALAT1 RNA in 7.5 mM sodium phosphate buffer at 10 °C. Shifts for both N^6-methylated A22 and unmodified RNA are shown. Shift differences greater than 0.02 ppm are highlighted in yellow.

		A-RNA	m^6A-RNA	Difference
A1	H1'	5.925	5.93	0.005
A1	H2	6.926	6.935	0.009
A1	H8	8.438	8.442	0.004
A2	H1'	5.909	5.911	0.002
A2	H2	7.734	7.737	0.003
A2	H8	7.978	7.981	0.003
C3	H1'	5.302	5.308	0.006
C3	H41	8.27	8.277	0.007
C3	H42	6.947	6.949	0.002
C3	H5	5.183	5.185	0.002
C3	H6	7.366	7.375	0.009
U4	H1'	5.491	5.493	0.002
U4	H3	13.899	13.904	0.005
U4	H5	5.307	5.309	0.002
U4	H6	7.66	7.665	0.005
U5	H1'	5.341	5.344	0.003
U5	H3	11.397	11.4	0.003
U5	H5	5.736	5.748	0.012
U5	H6	7.844	7.851	0.007
A6	H1'	5.8	5.804	0.004
A6	H2	6.78	6.78	0
A6	H8	8.226	8.23	0.004
A7	H1'	5.617	5.624	0.007
A7	H2	7.682[†]	7.689[†]	0.007
A7	H8	7.491	7.48	-0.011
U8	H1'	5.185	5.185	0
U8	H3	10.333	10.341	0.008
U8	H5	4.944	4.95	0.006
U8	H6	7.114	7.112	-0.002
G9	H1	13.392[†]	13.416[†]	0.024
G9	H21	6.326	6.356	0.03
G9	H22	8.761	8.791	0.03
U10	H3	13.873[†]	13.737[†]	-0.136
U11	H1'	5.51	5.529	0.019
U11	H3	11.842	11.914	0.072
U11	H6	7.889	7.89	0.001
U12	H1'	5.649	5.626	-0.023

U12	H3	11.856	11.871	0.015
G15	H1'	5.981	5.979	-0.002
G15	H8	8.268	8.266	-0.002
G20	H1	10.549	10.572	0.023
G20	H1'	5.676	5.71	0.034
G20	H2	6.167	6.183	0.016
G20	H8	7.782	7.771	-0.011
G21	H1	10.678	10.76	0.082
G21	H1'	5.651	5.628	-0.023
G21	H2	6.473	6.489	0.016
G21	H8	7.25	7.242	-0.008
A22	H1'	5.926	5.961	0.035
A22	H2	7.914[†]	7.834[†]	-0.08
m^6A22	H6		8.462[†]	
A22	H8	7.729	7.929	0.2
C23	H1'	5.304	5.262	-0.042
C23	H41	8.217	8.216	-0.001
C23	H42	7.049	7.259	0.21
C23	H5	5.253	5.345	0.092
C23	H6	7.262	7.186	-0.076
U24	H1'	5.505	5.515	0.01
U24	H3	10.598	10.609	0.011
U24	H5	5.45	5.462	0.012
U24	H6	7.623	7.635	0.012
U25	H1'	5.662	5.664	0.002
U25	H3	14.516[†]	14.537[†]	0.021
U25	H5	5.663	5.668	0.005
U25	H6	8.049	8.055	0.006
U26	H1'	5.732	5.735	0.003
U26	H2'	4.588	4.591	0.003
U26	H3	13.117[†]	13.124[†]	0.007
U26	H5	5.562	5.562	0
U26	H6	7.803	7.803	0
G27	H1	9.826	9.827	0.001
G27	H1'	5.682	5.684	0.002
G27	H2	6	5.993	-0.007
G27	H8	7.683	7.683	0
A28	H1'	5.793	5.792	-0.001
A28	H2	7.431	7.434	0.003
A28	H8	7.588	7.588	0
G29	H1	13.323	13.326	0.003

G29	H1'	5.532	5.534	0.002
G29	H21	6.274	6.276	0.002
G29	H22	8.502	8.505	0.003
G29	H8	7.036	7.038	0.002
U30	H1'	5.53	5.525	-0.005
U30	H3	14.34	14.342	0.002
U30	H6	7.682	7.683	0.001
U31	H1'	5.659	5.664	0.005
U31	H3	13.16	13.17	0.01
U31	H5	5.489	5.491	0.002
U31	H6	7.836	7.84	0.004
A32	H1'	5.864	5.869	0.005
A32	H2	7.206	7.207	0.001
A32	H8	8.102	8.104	0.002

[†]These assignments are different from those by Zhou et al.[30].

Table S8. The stacking nearest neighbor stability to be fit and the estimate of the fit. "ΔG°$_{37}$ Stacks with m^{6}A-U pairs" is the stability of the duplex (Table S1) minus the stability of Watson-Crick and G-U pair stacks and minus the stability of A-U end penalties, symmetry penalties, and initiation penalties. This is the term fit in the regression. The "ΔG°$_{37}$ Estimate" is estimated from the fit nearest neighbor parameters (Table S2). "Residual" is the difference between the two. The regression minimizes the sum of squares of residuals.

Sequence†:	ΔG°$_{37}$ Stacks with m^{6}A-U pairs (kcal/mol)	ΔG°$_{37}$ Estimate (kcal/mol)	Residual (kcal/mol)
66GGAGG/ UUCCUCC	-2.94	-1.76	-1.18
6CUGUC6/ UGACAGU	-3.14	-3.03	-0.11
GUC6CUG/ CAGUGAC	-3.32	-3.03	-0.29
6ACUAGUU$_2$	-1.81	-1.83	0.02
6UACGUAU$_2$	-1.32	-2.19	0.87
6UCUAG6U$_2$	-4.09	-4.35	0.26
ACU6UAGU$_2$	-2.76	-1.93	-0.83
ACUU6AGU$_2$	-1.06	-0.39	-0.67
ACUUA6GU$_2$	-4.93	-4.77	-0.16
AG6UAUCU$_2$	-5.78	-5.64	-0.14
AGUU66CU$_2$	-2.02	-2.55	0.53
AGUU6ACU$_2$	-0.47	-0.39	-0.08
AUCUAG6U$_2$	-6.36	-5.64	-0.73
CCUGU6GG$_2$	-3.68	-3.75	0.07
G66CGUUC$_2$	-7.38	-7.44	0.06
G6ACGUUC$_2$	-5.57	-5.28	-0.29
G6CGCGUU$_2$	-4.96	-4.22	-0.74
GA6CGUUC$_2$	-5.10	-5.24	0.14
GGUU6ACC$_2$	-0.16	-0.39	0.23
GUCUAG6U$_2$	-5.25	-4.82	-0.43
GUUCG6AC$_2$	-4.48	-5.28	0.80
U6CAUGUA$_2$	-4.77	-5.05	0.28
U6CCGGUG$_2$	-4.30	-4.23	-0.07
UAC6UGUA$_2$	-2.70	-2.93	0.24
UACAUGU6$_2$	-0.92	-1.47	0.55
UUCCGGA6$_2$	-1.63	-1.65	0.02
C6GUCGAUUG$_2$	-3.01	-2.54	-0.47
CGGUGC6UCG$_2$	-3.43	-3.86	0.43
CUGG6UUCAG$_2$	-1.03	-1.09	0.06
G6GAGCUUUC$_2$	-3.17	-3.50	0.33
G6GGAUCUUC$_2$	-3.36	-3.50	0.14
GAG6GCUUUC$_2$	-3.07	-3.75	0.68

Table S9. The number of occurrences of each stacking parameter in the set of fit helices.

Nearest Neighbor Stack:	Occurrences in Fitting Set:
5'6C3'<br>3'UG5'	14
5'UC3'<br>3'6G5'	18
5'6G3'<br>3'UC5'	7
5'UG3'<br>3'6C5'	8
5'6U3'<br>3'UA5'	6
5'6A3'<br>3'UU5'	12
5'UU3'<br>3'6A5'	6
5'UA3'<br>3'6U5'	6
5'6U3'<br>3'UG5'	4
5'6U3'<br>3'U65'	5
5'UG3'<br>3'6U5'	4
5'UU3'<br>3'6G5'	6
5'663'<br>3'UU5'	5
5'6G3'<br>3'UU5'	6
5'U63'<br>3'6U5'	4

Supplemental References:

1 Kierzek, R. *et al.* Polymer-supported synthesis and its application to test the nearest-neighbor model for duplex stability. *Biochemistry* **25**, 7840-7846 (1986).

2 He, L., Kierzek, R., SantaLucia, J., Jr., Walter, A. E. & Turner, D. H. Nearest-neighbor parameters for G·U mismatches. *Biochemistry* **30**, 11124-11132 (1991).

3 Xia, T. *et al.* Thermodynamic parameters for an expanded nearest-neighbor model for formation of RNA duplexes with Watson-Crick pairs. *Biochemistry* **37**, 14719-14735 (1998).

4 Ziomek, K., Kierzek, E., Biala, E. & Kierzek, R. The thermal stability of RNA duplexes containing modified base pairs placed at internal and terminal positions of the oligoribonucleotides. *Biophys Chem* **97**, 233-241 (2002).

5 Kierzek, E. & Kierzek, R. The thermodynamic stability of RNA duplexes and hairpins containing N6-alkyladenosines and 2-methylthio-N6-alkyladenosines. *Nucleic Acids Res* **31**, 4472-4480 (2003).

6 Chen, J. L. *et al.* Testing the nearest neighbor model for canonical RNA base pairs: revision of GU parameters. *Biochemistry* **51**, 3508-3522 (2012).

7 Bevington, P. R. & Robinson, D. K. *Data Reduction and Error Analysis for the Physical Sciences*. (McGraw Hill, 2003).

8 Mathews, D. H., Burkard, M. E., Freier, S. M., Wyatt, J. R. & Turner, D. H. Predicting oligonucleotide affinity to nucleic acid targets. *RNA* **5**, 1458-1469 (1999).

9 Sloma, M. F. & Mathews, D. H. Improving RNA secondary structure prediction with structure mapping data. *Methods Enzymol* **553**, 91-114 (2015).

10 Speek, M. & Lind, A. Structural analyses of *E. coli* 5S RNA fragments, their associates and complexes with proteins L18 and L25. *Nucleic Acids Res* **10**, 947-965 (1982).

11 Szymanski, M., Zielezinski, A., Barciszewski, J., Erdmann, V. A. & Karlowski, W. M. 5SRNAdb: an information resource for 5S ribosomal RNAs. *Nucleic Acids Res* **44**, D180-183 (2016).

12 Hopfinger, M. C., Kirkpatrick, C. C. & Znosko, B. M. Predictions and analyses of RNA nearest neighbor parameters for modified nucleotides. *Nucleic Acids Res* **48**, 8901-8913 (2020).

13 Mathews, D. H. Using an RNA secondary structure partition function to determine confidence in base pairs predicted by free energy minimization. *RNA* **10**, 1178-1190 (2004).

14 McCaskill, J. S. The equilibrium partition function and base pair probabilities for RNA secondary structure. *Biopolymers* **29**, 1105-1119 (1990).

15 Freier, S. M., Sinclair, A., Neilson, T. & Turner, D. H. Improved free energies for G-C base-pairs. *J Mol Biol* **185**, 645-647 (1985).

16 Freier, S. M. *et al.* Stability of XGCGCp, GCGCYp, and XGCGCYp helixes: an empirical estimate of the energetics of hydrogen bonds in nucleic acids. *Biochemistry* **25**, 3214-3219 (1986).

17 Petersheim, M. & Turner, D. H. Base-stacking and base-pairing contributions to helix stability: thermodynamics of double-helix formation with CCGG, CCGGp, CCGGAp, ACCGGp, CCGGUp, and ACCGGUp. *Biochemistry* **22**, 256-263 (1983).

18 Sugimoto, N., Kierzek, R. & Turner, D. H. Sequence dependence for the energetics of dangling ends and terminal base pairs in ribonucleic acid. *Biochemistry* **26**, 4554-4558 (1987).

19 Serra, M. J., Axenson, T. J. & Turner, D. H. A model for the stabilities of RNA hairpins based on a study of the sequence dependence of stability for hairpins of six nucleotides. *Biochemistry* **33**, 14289-14296 (1994).

20 Sugimoto, N., Kierzek, R. & Turner, D. H. Sequence dependence for the energetics of terminal mismatches in ribooligonucleotides. *Biochemistry* **26**, 4559-4562 (1987).

21 Freier, S. M., Burger, B. J., Alkema, D., Neilson, T. & Turner, D. H. Effects of 3' dangling end stacking on the stability of GGCC and CCGG double helices. *Biochemistry* **22**, 6198-6206 (1983).

22 Freier, S. M., Kierzek, R., Caruthers, M. H., Neilson, T. & Turner, D. H. Free energy contributions of G·U and other terminal mismatches to helix stability. *Biochemistry* **25**, 3209-3223 (1986).

23 Giese, M. R. *et al.* Stability of RNA hairpins closed by wobble base pairs. *Biochemistry* **37**, 1094-1100 (1998).

24 Fountain, M. A., Serra, M. J., Krugh, T. R. & Turner, D. H. Structural features of a six-nucleotide RNA hairpin loop found in ribosomal RNA. *Biochemistry* **35**, 6539-6548 (1996).

25 Walter, A. E., Wu, M. & Turner, D. H. The stability and structure of tandem GA mismatches in RNA depend on closing base pairs. *Biochemistry* **33**, 11349-11354 (1994).

26 SantaLucia, J., Jr., Kierzek, R. & Turner, D. H. Stabilities of consecutive A·C, C·C, G·G, U·C, and U·U mismatches in RNA internal loops: evidence for stable hydrogen-bonded U·U and $C \cdot C^+$ pairs. *Biochemistry* **30**, 8242-8251 (1991).

27 Liu, B. *et al.* A potentially abundant junctional RNA motif stabilized by m(6)A and Mg(2). *Nat Commun* **9**, 2761 (2018).

28 Gokhale, N. S. *et al.* N6-Methyladenosine in Flaviviridae Viral RNA Genomes Regulates Infection. *Cell Host Microbe* **20**, 654-665 (2016).

29 Zuber, J., Cabral, B. J., McFadyen, I., Mauger, D. M. & Mathews, D. H. Analysis of RNA Nearest Neighbor Parameters Reveals Interdependencies and Quantifies the Uncertainty in RNA Secondary Structure Prediction. *RNA* **24**, 1568-1582 (2018).

30 Zhou, K. I. *et al.* N(6)-Methyladenosine Modification in a Long Noncoding RNA Hairpin Predisposes Its Conformation to Protein Binding. *J Mol Biol* **428**, 822-833 (2016).
