# Supplementary material for: Secondary structure prediction for RNA sequences including N6-methyladenosine
Source: Nat Commun. 2022 Mar 11;13:1271. doi: 10.1038/s41467-022-28817-4 (PMC8917230; doi:10.1038/s41467-022-28817-4)
Supplement: Supplementary file 2 — Reporting Summary [file 41467_2022_28817_MOESM2_ESM.pdf]

nature research

Corresponding author(s): Elzbieta Kierzek
David H. Mathews

Last updated by author(s): 2021/8/18

Reporting Summary

Nature Research wishes to improve the reproducibility of the work that we publish. This form provides structure for consistency and transparency in reporting. For further information on Nature Research policies, see our Editorial Policies and the Editorial Policy Checklist.

Statistics

For all statistical analyses, confirm that the following items are present in the figure legend, table legend, main text, or Methods section.

n/a	Confirmed	
☐	☒	The exact sample size (n) for each experimental group/condition, given as a discrete number and unit of measurement
☐	☒	A statement on whether measurements were taken from distinct samples or whether the same sample was measured repeatedly
☒	☐	The statistical test(s) used AND whether they are one- or two-sided<br>*Only common tests should be described solely by name; describe more complex techniques in the Methods section.*
☒	☐	A description of all covariates tested
☒	☐	A description of any assumptions or corrections, such as tests of normality and adjustment for multiple comparisons
☐	☒	A full description of the statistical parameters including central tendency (e.g. means) or other basic estimates (e.g. regression coefficient) AND variation (e.g. standard deviation) or associated estimates of uncertainty (e.g. confidence intervals)
☒	☐	For null hypothesis testing, the test statistic (e.g. F, t, r) with confidence intervals, effect sizes, degrees of freedom and P value noted<br>*Give P values as exact values whenever suitable.*
☒	☐	For Bayesian analysis, information on the choice of priors and Markov chain Monte Carlo settings
☒	☐	For hierarchical and complex designs, identification of the appropriate level for tests and full reporting of outcomes
☒	☐	Estimates of effect sizes (e.g. Cohen's d, Pearson's r), indicating how they were calculated

Our web collection on statistics for biologists contains articles on many of the points above.

Software and code

Policy information about availability of computer code

Data collection	For optical melting data collection, Spectra Manager in Temperature Programming Measurement mode was used.<br>For gel scanning, Image Reader FLA-5000 v1.0 was used on a Fuji FLA-5100 Phosphorimager.<br>For NMR operation and data collection, we used VNMR version 6.1C.
Data analysis	Optical melting data was fit with MeltWin 3.5.<br>Nearest neighbor parameter fitting was performed with a Python script that will be provided to the reviewers.<br>Chemical mapping data from the gel scan was analyzed using SAFA 1.1.<br>Secondary structure prediction was performed with RNAstructure 6.3, which is freely available under a the GNU license. This will also be provided to the reviewers with sample data.<br>Spectra from 2D NMR were processed and assigned with NMRpipe 8.2 and NMRFAM-SPARKY 1.2.

For manuscripts utilizing custom algorithms or software that are central to the research but not yet described in published literature, software must be made available to editors and reviewers. We strongly encourage code deposition in a community repository (e.g. GitHub). See the Nature Research guidelines for submitting code & software for further information.

Data

Policy information about availability of data

All manuscripts must include a data availability statement. This statement should provide the following information, where applicable:
- Accession codes, unique identifiers, or web links for publicly available datasets
- A list of figures that have associated raw data
- A description of any restrictions on data availability

The results of the optical melting experiments needed to reproduce the results in this study are provided in the Supplementary Materials (Tables S1 and S3). The

raw data are available from the corresponding authors upon request. The nearest neighbor parameters are available as Supplementary Materials and as part of the RNAstructure software package.

Field-specific reporting

Please select the one below that is the best fit for your research. If you are not sure, read the appropriate sections before making your selection.

☒ Life sciences ☐ Behavioural & social sciences ☐ Ecological, evolutionary & environmental sciences

For a reference copy of the document with all sections, see nature.com/documents/nr-reporting-summary-flat.pdf

Life sciences study design

All studies must disclose on these points even when the disclosure is negative.

Sample size	No sample size calculation was performed. The number of optical melting experiments that was needed was based on prior work that estimated nearest neighbor parameters.<br>For determination of nearest neighbor parameters, the error estimates support that an adequate number of optical melting experiments was performed.
Data exclusions	No data were excluded from analyses.
Replication	Optical melting data were collected over a range of sample concentrations, for a total of nine concentrations for each model structure.<br>Chemical mapping experiments were performed in duplicate.
Randomization	Randomization was not applicable for this study. Experiments were in vitro and quantitative.
Blinding	Blinding was not applicable for this study. Experiments are quantitative. We also tested the nearest neighbor parameters with additional quantitative experiments.

Reporting for specific materials, systems and methods

We require information from authors about some types of materials, experimental systems and methods used in many studies. Here, indicate whether each material, system or method listed is relevant to your study. If you are not sure if a list item applies to your research, read the appropriate section before selecting a response.

Materials & experimental systems

n/a	Involved in the study	
☒	☐	Antibodies
☒	☐	Eukaryotic cell lines
☒	☐	Palaeontology and archaeology
☒	☐	Animals and other organisms
☒	☐	Human research participants
☒	☐	Clinical data
☒	☐	Dual use research of concern

Methods

n/a	Involved in the study	
☒	☐	ChIP-seq
☒	☐	Flow cytometry
☒	☐	MRI-based neuroimaging
