# Supplementary material for: Secondary structure prediction for RNA sequences including N6-methyladenosine
Source: Nat Commun. 2022 Mar 11;13:1271. doi: 10.1038/s41467-022-28817-4 (PMC8917230; doi:10.1038/s41467-022-28817-4)
Supplement: Supplementary file 4 — Description of Additional Supplementary Files [file 41467_2022_28817_MOESM4_ESM.pdf]

Title: Supporting Software 1
Description: Python code and input dataset for fitting helical stack nearest neighbor parameters for stacks that include m^6A-U base pairs.

Title: Supporting Software 2
Description: Plain text nearest neighbor parameter files that include m^6A nucleotides.
